# Selected predictors of parental satisfaction with child nursing care in paediatric wards in Poland—Cross-sectional study

**DOI:** 10.1371/journal.pone.0260504

**Published:** 2021-11-19

**Authors:** Agnieszka Kruszecka-Krówka, Grażyna Cepuch, Agnieszka Gniadek, Ewa Smoleń, Krystyna Piskorz-Ogórek, Agnieszka Micek

**Affiliations:** 1 Nursing and Midwifery Institute, Faculty of Health Sciences, Jagiellonian University Medical College, Krakow, Poland; 2 Chair and Department of Management in Nursing, Faculty of Health Sciences, Medical University of Lublin, Lublin, Poland; 3 Department of Nursing, School of Public Health, Medical College, University of Warmia and Mazury in Olsztyn, Olsztyn, Poland; Kaohsuing Medical University Hospital, TAIWAN

## Abstract

**Background:**

Assessment of parental satisfaction with child nursing is the key issue in evaluation of the care quality, enabling the adjustment of the services provided to the needs and expectations of recipients, and thus ensuring safety and achieving better long-term health effects.

**Aim:**

Assessment of parental satisfaction with child nursing in paediatric wards including its determinants.

**Material and methods:**

The study covered 1030 parents of children hospitalised in paediatric and surgical wards of seven hospitals of different levels of health security in Poland. The Polish adaptation of the Empathic standardised questionnaire for assessment of the level of parents’ satisfaction with nursing care, developed by Latour et al. and the self-constructed summary of socio-demographic data were applied in the study.

**Results:**

More than 90% of respondents expressed high level of satisfaction with nurses’ Availability, the lowest, but still high score of respondents’ satisfaction was observed for Parental Participation. The highest satisfaction was observed among the parents of children at the preschool, early school and puberty stage, admitted to the hospital on the elective basis, referred for diagnostic assessment and with the length of hospital stay less than 7 and longer than 28 days. Achieving preschool age was the strongest factor which increased assessment of satisfaction in most domains.

**Conclusions:**

There is a need for optimising nursing care especially in the area of parental participation. The nursing care’ quality improvement plan in paediatric departments should focus particularly on early childhood patients and their parents who are the most critical in satisfaction’ assessment.

## Introduction

Paediatric patients require a specific hospital environment, personalised approach of the medical team and parents’ participation in care at each step of the therapeutic process [1]. Thus, to make sure a child is provided with high-quality nursing services during hospitalisation, it is necessary to focus on a patient’s health and developmental issues and to consider the aspects of family and the social context while planning particular services. Quality is one of the most important features of health care and the key determinant of satisfaction with services [2]. For patients and their parents/families, the quality of health services, including nursing care, primarily means subjective satisfaction with care provided [3]. When a child is hospitalised, the level of satisfaction with services received is mostly assessed by a parent who is the main caregiver and participates in the care and related decision-making [4].

Evaluation of satisfaction with nursing care is the key issue of the overall medical service quality and it is perceived as a difference between patients’ or their families’ expectations about nursing services and the perception of care that was actually provided. It is an integral part of holistic nursing care that gives a potential for discovering patients’ needs, expectations, service-related opinions and views with regard to respecting patients’ rights during hospitalisation [3–5]. It also enables assessment of the situation and optimisation of services provided [2, 5, 6], thus inducing increased profitability and competitiveness of the health care institution as well as its promotion among potential patients [5–7]. A satisfied patient/parent more frequently accepts the employed treatment management plan, which consequently may lead to healthier behaviours after hospital discharge [5–7]. Moreover, high satisfaction with care determines professional satisfaction among the medical staff [2].

Parents’ expectations with regard to nursing care can be conditioned by developmental issues and the type of clinical diagnosis as well as by the disease course, child’s clinical status or his/her subjective perception [7–9], the length of hospital stay, previous experience of accompanying the child during hospitalisation [8, 10] and the final treatment outcomes [11]. In addition, a relationship between the level of quality of nursing care/satisfaction with care and the system conditions and healthcare organisation is observed [12]. Also impact of cultural or racial backgrounds [11, 13] as well as individual factors, including selected socio-demographic variables of the subjects [2, 4, 7, 14, 15] and their emotional state [7, 16] is suggested.

The aim of the study is the assessment of parental satisfaction with nursing care in paediatric wards including its potential determinants. Knowledge of parents’ opinions on nursing care in selected Polish paediatric hospitals and assessment of satisfaction predictors with the use of a standardised research instrument will help with better service planning and their adjustment to the recipients’ needs, resulting in higher satisfaction with services offered and achievement of improved future long-term health outcomes in children.

## Materials and methods

### Study design

The cross-sectional study was designed to evaluate parental satisfaction with child nursing in paediatric wards including its potential determinants. We used the STROBE cross sectional checklist when writing our report [17]. Written consent for participation was obtained prior to data collection. The privacy and confidentiality of participants was strictly protected. All the information provided by each participant was coded by a number that does not directly identify any individual and all identifying information was coded and removed from all non-numerical data to make it impossible for anyone but the experimenter to identify any individual. The study was carried out in accordance with the ethical principles of the Helsinki Declaration. The protocol of the study was approved by the Bioethics Committee of the Jagiellonian University (No. 122.6120.254.2016).

#### Patient and public involvement

While patients were not involved in the study, their parents were the only source of information on the quality of nursing care from the perspective of the service users. For this reason, assessment of satisfaction with nursing care obtained in the study is particularly valuable in the context of the interpersonal aspect of services. The study findings can be the basis for development and implementation of the training plan for nursing teams in paediatric hospitals with the aim of optimisation and personalisation of nursing care.

#### Description of research tools

To assess parental satisfaction with child nursing, the method of diagnostic survey was applied including the following survey questionnaires:

1. The EMPATHIC standardised parent/caregiver satisfaction-with-nursing-care questionnaire, developed by Latour et al. [18] and adapted to the Polish conditions by Smoleń and Ksykiewicz Dorota [19].
2. A socio-demographic summary including the parents’ details (sex, age, place of residence, education and the number of children) and variables regarding the child and the hospitalisation process (the child’s age, reason of hospitalisation, co-morbidities, hospital admission type, length of stay and the previous hospitalisation experience within the past 12 months). The sociodemographic variables as well as variables regarding the child and the hospitalisation process were used in the models as predictors of parents’ satisfaction.

The standardised questionnaire for assessment of the parent/caregiver satisfaction with nursing care consists of five major domains: Information, Care and Treatment, Availability, Parental Participation and Professional Attitude (Professionalism), containing 2 to 19 detailed criteria. Each of the detailed criteria was assessed by the parents using the 5-point Likert scale where “1” denoted “I am very dissatisfied” and “5” meant “I am very satisfied”. The overall score of the parental satisfaction with child nursing was a mean of all scores for each of the major domains. The score for the major domains was a mean of the scores for the assigned detailed criteria. The satisfaction scores were expressed as point values with the accuracy of two decimal places.

High reliability rates were obtained for the tool. Cronbach’s alpha-coefficient for overall satisfaction with care was 0.965, while for the main criteria: 0.853—Information, 0.786—Care and Treatment, 0.926 –Availability, 0.786—Parental Participation and 0.909—Professionalism [19].

The score of 1.00 to 2.49 denoted a low level of satisfaction with nursing care (overall, for the main domains and for each detailed criterion). The score of 2.50 to 3.99 corresponds to the medium level of satisfaction with care. A high level of satisfaction with nursing care is considered to score 4.00 to 5.00 points.

Due to extensive research material, the scores only regarding the overall parental satisfaction and satisfaction regarding the main domains were presented: Information, Care and Treatment, Availability, Parental Participation and Professionalism.

A division of children into age groups was created in accordance with the paediatric classification corresponding to the developmental stages, while the age of parents was assigned to one of three categories: up to 30 years of age, 31–40 years, above 40 years of age. The duration of hospitalisation was assigned to one of categories: up to 7 days, 8–14 days, 15–21 days, 22–28 days and over 28 days. The categories for the age of parents and the length of hospitalisation of children were adopted after Smoleń and Ksykiewicz-Dorota [4, 10] to enable the results of the current study to be compared with the results of other scientific studies carried out in Poland.

### Sample & setting

An a priori calculated sample size of 1030 individuals was planned to could estimate the proportion of parents with high satisfaction with child nursing in the paediatric wards controlling the probability of a Type I and Type II errors at level 0.05 and 0.10, respectively and assuring the precision of estimation of 0.05. The study was conducted on a sample of parents of children hospitalised at surgical and paediatric wards of 7 leading Polish paediatric sites in Lesser Poland (Krakow I, Krakow II), Subcarpathian (Sanok, Krosno, Rzeszów, Brzozów) and Warmian-Masurian Voivodhips (Olsztyn), between 2017 and 2020. The selection of hospitals for the study was deliberate. The study was conducted in hospitals participating in the project of satisfaction with nursing care assessment before [2, 4, 10], in order to compare the obtained results with the findings of other authors.

Considering the shift work system intended for most nurses in Polish hospitals (mainly 12-hour day and night shifts) as well as the mean 4-day and 3-day hospital stay length at paediatric and paediatric surgery departments, respectively [20], a criterion of at least 3-day hospitalisation was set as one of the inclusion requirements. The other inclusion criteria were as follows: permanent or temporary, but not shorter than 12 hours per day, presence of the parent with the child during the hospital stay, the voluntary consent to participate in the study and Polish nationality or other nationality, but good knowledge of Polish in speech and writing. The exclusion criterion was a child’s end-of-life stage. The study included all parents who met the inclusion criteria and consented to participate in the study.

### Data collection

The data was collected during direct meetings with the parents. The nursing staff members were not involved in the study. Having received the hospital discharge summary, one of the parents (being the primary caregiver during the child’s hospital stay) completed survey questionnaires. Participation in the study was voluntary and anonymous. All parents gave their informed written consent to participate in the study. Parents were informed about the possibility of withdrawing from participation in the study at any stage of completing the questionnaire, and it was ensured that both the refusal to consent to participate in the study, as well as the resignation from participation in the study will not affect the quality of care for the child.

#### Statistical analysis

Due to deviation from normality, the satisfaction in specific domains and in overall were characterised by the measures of position (median and quartiles). Further, to assure robustness of the findings considering the high skewness of data, the non-parametric tests were used to examine differences between the categories of demographic and hospital variables. The comparisons between two groups were performed by Wilcoxon’s rank sum test, and in the case of more groups the ANOVA on ranks Kruskal-Wallis test was applied. Hierarchical linear regression analysis was utilized to explore the association of satisfaction in specific domains and in overall with multiple factors. To minimize the potential sources of bias we performed multivariable analyses controlling for the main possible confounders but not introducing in regression models unnecessarily redundant variables which could reduce the power. To check robustness of the findings, in the sensitivity analysis framework, a couple of models was fitted to adjust for a different sets of confounding variables. Most hospitals show a similar profile of each type of satisfaction across different categories of included variables therefore we assumed only random intercept per hospital and the remaining covariates were entered to a model as fixed effects. General linear mixed models was fitted separately for each domain of satisfaction and in overall. For each model an analogue of R^2^ goodness-of-fit metric was calculated and the total variance was decomposed to fraction explained by random effects, fixed effects and to a residual part. The normality assumptions of random effects and residuals was checked graphically in QQ-plots (S1 and S2 Figs). The statistical analyses were performed using the R Software for Windows (R Foundation for Statistical Computing, Vienna, Austria, version 4.0.4). Two-tailed significance level was set at p<0.05.

## Results

### Characteristics of the study participants

The study group consisted of 1030 parents. Women accounted for 87.30% (n = 899) of the respondents. The median age of parents was 33.00 (Q1 = 29; Q3 = 39). The median children’s age amounted to 3.13 years (Q1 = 0.92; Q3 = 7.31) and 75% (n = 772) of the hospital admissions were emergency cases. About 82.30% (n = 848) of the children were hospitalised in the paediatric (non-surgical) wards. The main admission reason was sudden illness observed in 72.30% (n = 745) and 44.90% (n = 462) of the hospital stays lasted for up to 7 days (n = 462). The median duration of hospitalisation was 8 days (Q1 = 5; Q3 = 15). Table 1 presents details of the study group characteristics.

**Table 1:** Characteristics of the study participants (N = 1030).

Selected variables regarding the parents, children and the hospitalisation process		[n]	[%]
**Parents’ sex**	female	899	**87.30**
	male	131	12.70
**Parents’ age**	up to 30 years of age	325	31.60
	31 to 40 years	522	**50.70**
	above 40 years of age	156	15.10
	no data available	27	2.60
**Education**	higher	622	**60.40**
	other than higher	408	39.50
**Place of residence**	city/town	425	41.30
	village	605	**58.70**
**Number of children**	one	335	32.50
	two	480	**46.60**
	more than two	215	20.90
**Child’s developmental stage**	newborn and infant	298	**28.90**
	toddler	212	20.60
	preschool	192	18.60
	early school	228	22.10
	puberty	100	9.70
**Child’ hospital admission**	emergency	772	**75.00**
	elective	258	25.00
**Reason of the admission**	chronic disease exacerbation	151	14.70
	sudden illness	745	**72.30**
	diagnostic assessment and other	134	13.00
**Co-morbidities**	yes	149	14.50
	no	881	**85.50**
**Length of hospital stay**	up to 7 days	462	**44.90**
	8–14 days	304	29.50
	15–21 days	170	16.50
	22–28 days	65	6.30
	over 28 days	29	2.80
**Hospital stay**	first	757	**73.50**
	further	273	26.50

**Notes**: Complete participant descriptive.

### Parental satisfaction with child nursing

**Fig 1:**
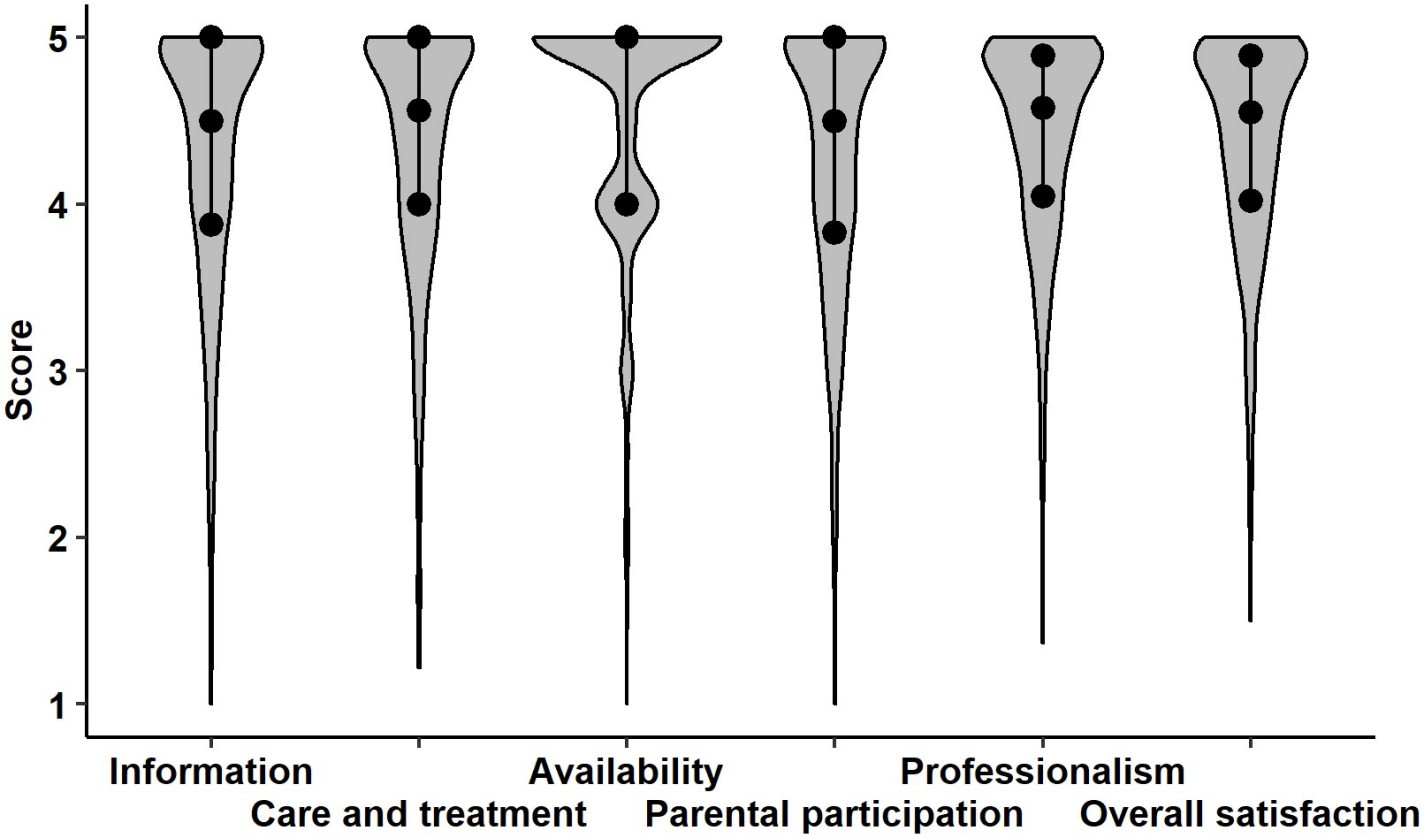
The entire distribution of satisfaction by each domain and in total. Notes: The points in the middle of violin and black intervals show median and interquartile range, respectively. Wider sections of the violin plot represent a higher probability that members of the population will take on the given value; the skinnier sections represent a lower probability.

About 76% of parents presented high level of overall satisfaction with nursing care (Q2 = 4.55; Q1 = 4.02; Q3 = 4.89). Similar results were obtained for the individual major satisfaction’ criteria: Information, Care and Treatment, Availability, Parental Participation and Professionalism. Parents expressed the highest level of satisfaction with nursing care in Availability (Q2 = 5.00; Q1 = 4.00; Q3 = 5.00), in which nearly 91% of them declared satisfaction level equal to or greater than 4 points. The lowest, but still high score of respondents’ satisfaction was observed for Parental participation (Q2 = 4.50; Q1 = 3.83; Q3 = 5.00), where 72% of parents were great pleased. The entire distribution of satisfaction by each domain and in total was depicted in Table 2 and in Fig 1.

**Table 2:** Parental satisfaction with nursing care in paediatric wards.

Variable	n	Q2 (Q1-Q3)	min-max	score ≥4.00, n (%)
Information	1030	4.50 (3.88; 5.00)	1.00–5.00	770 (74.76)
Care and Treatment	1030	4.56 (4.00; 5.00)	1.22–5.00	778 (75.53)
Availability	1030	**5.00 (4.00; 5.00)**	1.00–5.00	**933 (90.58)**
Parental Participation	1030	**4.50 (3.83; 5.00)**	1.00–5.00	**749 (72.72)**
Professionalism	1030	4.58 (4.05; 4.89)	1.37–5.00	811 (78.74)
**Overall satisfaction**	1030	4.55 (4.02; 4.89)	1.50–5.00	783 (76.02)

Q2, Q1, Q3 –quartiles.

There was a significant difference in satisfaction (overall and in each criterion) between all considered demographic factors except of parents’ sex and place of residence. Higher level of satisfaction (in all criteria and in overall) was declared by parents who did not have high education, were over thirties, whose children were older (preschool, early school, puberty) and had at least one siblings. Mothers compered to fathers declared higher satisfaction level in Availability domain. Place of residence was not associated with evaluation of any kind of satisfaction’ domain (Table 3).

**Table 3:** Parental satisfaction in each domain and in total by demographic characteristics.

Variable	n (%)	Information	Care and Treatment	Availability	Parental Participation	Professionalism	Overall satisfaction
		Q2 (Q1-Q3)	Q2 (Q1-Q3)	Q2 (Q1-Q3)	Q2 (Q1-Q3)	Q2 (Q1-Q3)	Q2 (Q1-Q3)
**Child’s developmental stage**							
newborn and infant	298 (28.90)	4.38 (3.63–4.88)**	4.44 (3.78–5.00)**	5.00 (4–5)*	4.42 (3.67–5.00)**	4.58 (3.95–4.89)**	4.42 (3.89–4.89)**
toddler	212 (20.60)	4.38 (3.63–5.00)	4.33 (3.67–5.00)	5.00 (4–5)	4.33 (3.67–4.83)	4.37 (3.79–4.79)	4.35 (3.79–4.77)
preschool	192 (18.60)	4.63 (4.13–5.00)	4.67 (4.11–5.00)	5.00 (4.5–5)	4.67 (4.00–5.00)	4.71 (4.32–4.89)	4.62 (4.26–4.91)
early school	228 (22.10)	4.75 (4.00–5.00)	4.67 (4.22–5.00)	5.00 (4.5–5)	4.67 (4.00–5.00)	4.71 (4.32–4.95)	4.66 (4.23–4.91)
puberty	100 (9.70)	4.75 (4.00–5.00)	4.67 (4.00–5.00)	5.00 (4–5)	4.58 (4.00–5.00)	4.61 (4.05–4.95)	4.62 (4.08–4.95)
**Number of children**							
one	335 (32.50)	4.50 (3.75–5.00)*	4.44 (3.78–4.95)**	5.00 (4–5)	4.33 (3.83–5.00)*	4.53 (3.95–4.84)**	4.43 (3.88–4.82)**
two	480 (46.60)	4.56 (4.00–5.00)	4.56 (4.00–5.00)	5.00 (4–5)	4.50 (3.83–5.00)	4.63 (4.11–4.89)	4.57 (4.05–4.91)
more than two	215 (20.90)	4.63 (4.00–5.00)	4.67 (4.22–5.00)	5.00 (4.25–5)	4.67 (3.92–5.00)	4.68 (4.26–4.95)	4.64 (4.23–4.95)
**Parents’ sex**							
female	899 (87.30)	4.50 (4.00–5.00)	4.56 (4.00–5.00)	5.00 (4–5)*	4.50 (3.83–5.00)	4.63 (4.08–4.89)	4.55 (4.02–4.89)
male	131 (12.70)	4.50 (3.81–5.00)	4.44 (3.89–5.00)	5.00 (4–5)	4.33 (3.67–5.00)	4.53 (4.00–4.84)	4.48 (3.99–4.81)
**Education**							
other than higher	408 (39.60)	4.63 (4.00–5.00)*	4.67 (4.11–5.00)**	5.00 (4–5)*	4.67 (4.00–5.00)*	4.68 (4.11–4.95)*	4.61 (4.11–4.93)**
higher	622 (60.40)	4.50 (3.75–5.00)	4.56 (3.89–5.00)	5.00 (4–5)	4.50 (3.83–5.00)	4.53 (4.05–4.84)	4.48 (3.95–4.84)
**Place of residence**							
city/town	605 (58.70)	4.50 (3.88–5.00)	4.56 (3.89–5.00)	5.00 (4.00–5.00)	4.50 (3.83–5.00)	4.58 (4.05–4.89)	4.52 (4.00–4.89)
village	425 (41.30)	4.63 (4.00–5.00)	4.56 (4.00–5.00)	5.00 (4.00–5.00)	4.50 (3.83–50)	4.58 (4.05–4.89)	4.57 (4.05–4.91)
**Parents’ age**							
up to 30 years of age	326 (31.70)	4.5 (3.75–5.00)*	4.44 (3.89–5.00)*	5.00 (4.00–5.00)	4.33 (3.67–50)	4.53 (3.96–4.84)*	4.43 (3.89–4.88)*
31 to 40 years	521 (50.60)	4.63 (4.00–5.00)	4.67 (4.00–5.00)	5.00 (4.00–5.00)	4.50 (3.83–5.00)	4.68 (4.16–4.89)	4.59 (4.09–4.89)
above 40 years of age	158 (15.30)	4.63 (4.00–5.00)	4.56 (4.00–5.00)	5.00 (4.00–5.00)	4.50 (4.00–5.00)	4.61 (4.12–4.89)	4.62 (4.07–4.91)

*p<0.05, **p<0.001 based on ANOVA on ranks Kruskal-Wallis test or Wilcoxon’s rank sum test

#### Parental satisfaction with nursing care vs. the hospital health care coverage level, ward type, previous hospitalisation experience and hospital admission type

**Fig 2:**
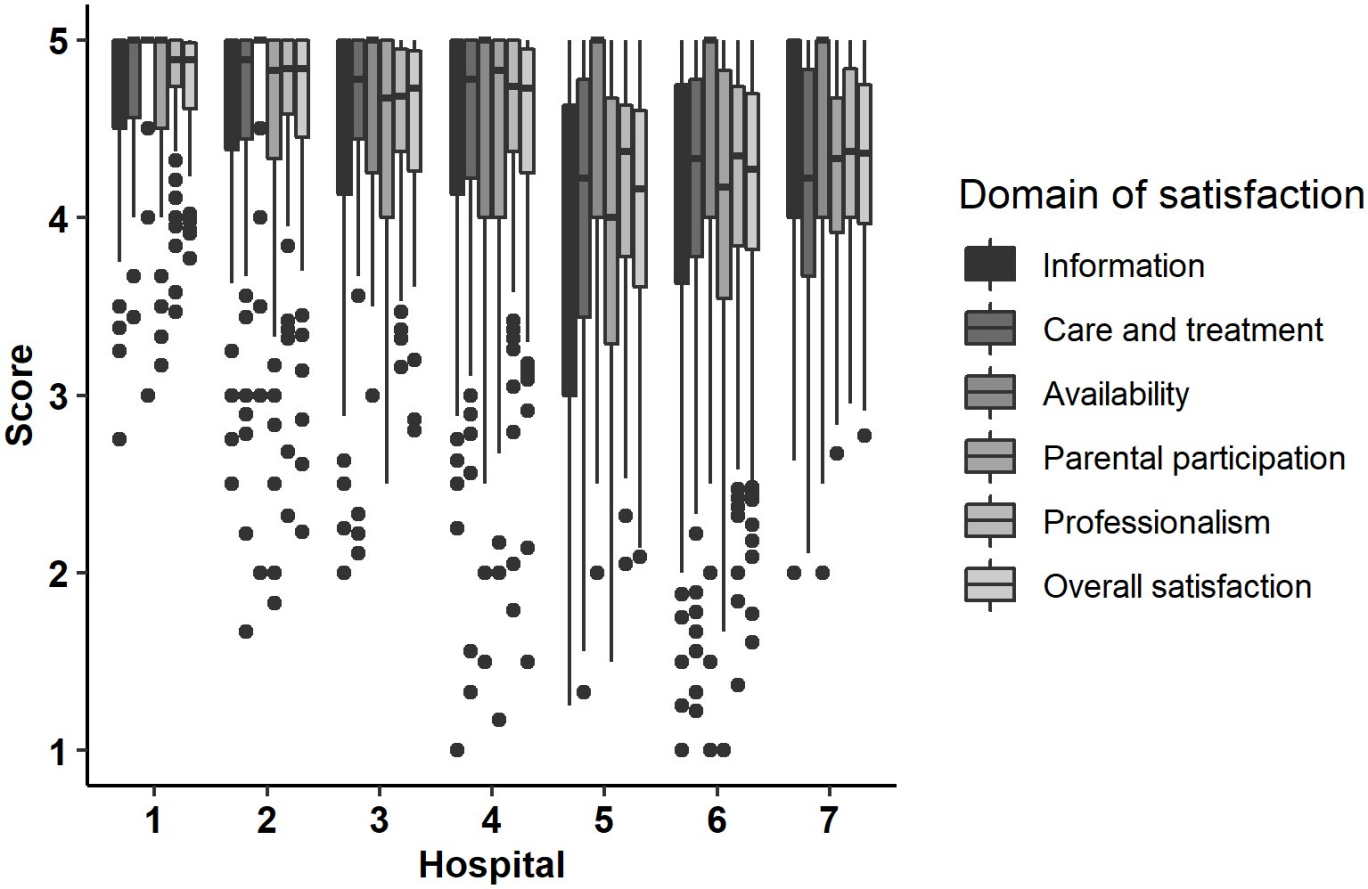
Trends of satisfaction (by domains and overall) across hospitals included in a study.

In all hospitals most parents estimated each domain of satisfaction very high. Only in Rzeszów, Kraków I and Kraków II the major criteria such as Information, Care and Treatment and Parental Participation was assessed slightly lower by respondents. The highest satisfaction in all criteria were observed in Sanok and Krosno, the lowest in Rzeszów. Distribution of satisfaction by domain and in overall across hospital is presented in Fig 2 and Table 4.

**Table 4:** Parental satisfaction in each domain and in total by hospital characteristics.

Variable	n (%)	Information	Care and Treatment	Availability	Parental Participation	Professionalism	Overall satisfaction
		Q2 (Q1-Q3)	Q2 (Q1-Q3)	Q2 (Q1-Q3)	Q2 (Q1-Q3)	Q2 (Q1-Q3)	Q2 (Q1-Q3)
**Hospital**							
Sanok	100 (9.70)	4.88 (4.50–5.00)**	5 (4.56–5.00)**	5.00 (5.00–5.00)**	5.00 (4.50–5.00)**	4.89 (4.74–5.00)**	4.89 (4.61–4.99)**
Krosno	101 (9.80)	4.88 (4.38–5.00)	4.89 (4.44–5.00)	5.00 (5.00–5.00)	4.83 (4.33–5.00)	4.84 (4.58–5.00)	4.84 (4.45–5.00)
Brzozów	99 (9.60)	4.63 (4.13–5.00)	4.78 (4.44–5.00)	5.00 (4.25–5.00)	4.67 (4.00–5.00)	4.68 (4.37–4.95)	4.73 (4.26–4.94)
Olsztyn	189 (18.30)	4.75 (4.13–5.00)	4.78 (4.22–5.00)	5.00 (4.00–5.00)	4.83 (4.00–5.00)	4.74 (4.37–5.00)	4.73 (4.25–4.95)
Rzeszów	100 (9.70)	3.75 (3.00–4.63)	4.22 (3.44–4.78)	5.00 (4.00–5.00)	4.00 (3.29–4.67)	4.37 (3.78–4.63)	4.16 (3.61–4.60)
Kraków I	346 (33.60)	4.25 (3.63–4.75)	4.33 (3.78–4.78)	5.00 (4.00–5.00)	4.17 (3.54–4.83)	4.35 (3.84–4.74)	4.27 (3.82–4.70)
Kraków II	95 (9.20)	4.38 (4.00–5.00)	4.22 (3.67–4.83)	5.00 (4.00–5.00)	4.33 (3.92–4.67)	4.37 (4.00–4.84)	4.36 (3.96–4.75)
**The levels of health care coverage**							
Nationwide hospital	346 (33.60)	4.25 (3.63–4.75)**	4.33 (3.78–4.78)**	5.00 (4.00–5.00)**	4.17 (3.54–4.83)**	4.35 (3.84–4.74)**	4.27 (3.82–4.70)**
Children’s hospital	284 (27.60)	4.75 (4.00–5.00)	4.56 (4.00–5.00)	5.00 (4.00–5.00)	4.58 (4.00–5.00)	4.65 (4.16–4.95)	4.61 (4.11–4.91)
2nd level hospital	100 (9.70)	4.69 (4.13–5.00)	4.78 (4.44–5.00)	5.00 (4.38–5.00)	4.67 (4.00–5.00)	4.68 (4.37–4.95)	4.73 (4.26–4.95)
3rd level hospital	199 (19.30)	4.50 (3.63–5.00)	4.67 (4.00–5.00)	5.00 (5.00–5.00)	4.50 (3.67–5.00)	4.68 (4.11–4.89)	4.61 (4.00–4.91)
Pulmonology/Oncological hospital	101 (9.80)	4.88 (4.38–5.00)	4.89 (4.44–5.00)	5.00 (5.00–5.00)	4.83 (4.33–5.00)	4.84 (4.58–5.00)	4.84 (4.45–5.00)
**Type of wards**							
paediatric	848 (82.30)	4.50 (3.97–5.00)	4.56 (4.00–5.00)	5.00 (4.00–5.00)	4.50 (3.83–5.00)	4.58 (4.11–4.89)	4.55 (4.04–4.89)
surgical	182 (17.70)	4.56 (3.88–5.00)	4.50 (3.89–5.00)	5.00 (4.00–5.00)	4.67 (3.67–5.00)	4.55 (3.95–4.88)	4.52 (3.95–4.91)
**Hospital stay**							
first	755 (73.30)	4.50 (3.94–5.00)	4.56 (4.00–5.00)	5.00 (4.00–5.00)	4.50 (3.83–5.00)	4.58 (4.11–4.89)	4.55 (4.02–4.9)
further	275 (26.70)	4.50 (3.88–5.00)	4.56 (3.89–5.00)	5.00 (4.00–5.00)	4.33 (3.83–5.00)	4.58 (4.05–4.89)	4.52 (4.01–4.86)
**Co-morbidities**							
yes	149 (14.50)	4.63 (3.88–5.00)	4.56 (4.00–5.00)	5.00 (4.00–5.00)	4.50 (3.83–5.00)	4.63 (4.11–4.89)	4.64 (4.05–4.91)
no	881 (85.50)	4.50 (3.88–5.00)	4.56 (4.00–5.00)	5.00 (4.00–5.00)	4.50 (3.83–5.00)	4.58 (4.05–4.89)	4.55 (4.00–4.89)
**Child’ hospital admission**							
emergency	772 (75.00)	4.50 (3.88–5.00)*	4.56 (4.00–5.00)	5.00 (4.00–5.00)	4.50 (3.83–5.00)*	4.58 (4.11–4.89)	4.52 (3.98–4.89)
elective	258 (25.00)	4.63 (4.00–5.00)	4.56 (4.00–5.00)	5.00 (4.00–5.00)	4.67 (4.00–5.00)	4.58 (4.05–4.95)	4.60 (4.05–4.91)
**Reason of the admission**							
chronic disease exacerbation	151 (14.70)	4.63 (4.00–5.00)*	4.56 (4.00–5.00)	5.00 (4.00–5.00)	4.50 (4.00–5.00)*	4.68 (4.21–4.89)*	4.61 (4.07–4.89)*
sudden illness	745 (72.30)	4.50 (3.88–5.00)	4.56 (3.89–5.00)	5.00 (4.00–5.00)	4.50 (3.83–5.00)	4.58 (4.00–4.89)	4.50 (3.98–4.89)
diagnostic assessment and other	134 (13.00)	4.69 (4.00–5.00)	4.67 (4.00–5.00)	5.00 (4.50–5.00)	4.67 (4.00–5.00)	4.68 (4.26–4.95)	4.68 (4.18–4.92)
**Length of hospital stay**							
up to 7 days	462 (44.90)	4.75 (4.00–5.00)**	4.78 (4.22–5.00)**	5.00 (4.50–5.00)**	4.67 (4.00–5.00)**	4.74 (4.22–4.95)**	4.68 (4.20–4.93)**
8–14 days	303 (29.40)	4.50 (3.75–4.94)	4.44 (3.89–4.95)	5.00 (4.00–5.00)	4.33 (3.83–4.83)	4.53 (4.03–4.87)	4.45 (3.95–4.82)
15–21 days	171 (16.60)	4.25 (3.63–4.88)	4.33 (3.78–4.78)	5.00 (4.00–5.00)	4.17 (3.67–4.83)	4.42 (3.95–4.79)	4.34 (3.89–4.74)
22–28 days	65 (6.30)	4.38 (3.75–4.88)	4.44 (3.78–4.78)	5.00 (4.00–5.00)	4.33 (3.67–4.83)	4.37 (3.89–4.79)	4.32 (3.89–4.73)
over 28 days	29 (2.80)	4.88 (4.50–5.00)	4.78 (4.22–5.00)	5.00 (4.00–5.00)	4.83 (4.00–5.00)	4.74 (4.42–5.00)	4.73 (4.18–4.98)

*p<0.05, **p<0.001 based on ANOVA on ranks Kruskal-Wallis test or Wilcoxon’s rank sum test.

Statistically significant differences between the overall parental satisfaction, satisfaction scores for all main domains (Information, Care and Treatment, Availability, Parental Participation and Professionalism) and the hospital health care coverage level were demonstrated. The parents of paediatric patients staying in the oncological hospital expressed the highest overall satisfaction with care and the highest satisfaction scores for all main domains. The parents of the nationwide hospital patients demonstrated both the lowest overall satisfaction with nursing care and the lowest satisfaction score for each of the main criteria.

The parents of children admitted to the hospital on the elective basis showed a statistically significant higher level of satisfaction with care regarding the main domains: Information and Parental Participation.

The ward type did not differentiate the overall parental satisfaction or the satisfaction scores for the domains: Information, Care and Treatment, Availability, Parental Participation and Professionalism. Statistically significant differences were not observed for both the overall parental satisfaction with care and satisfaction scores for the main domains regarding the first-time and further children’s admissions (Table 4).

#### Parental satisfaction with care vs. children’s developmental stage as well as the reason and length of hospital stay

**Fig 3:**
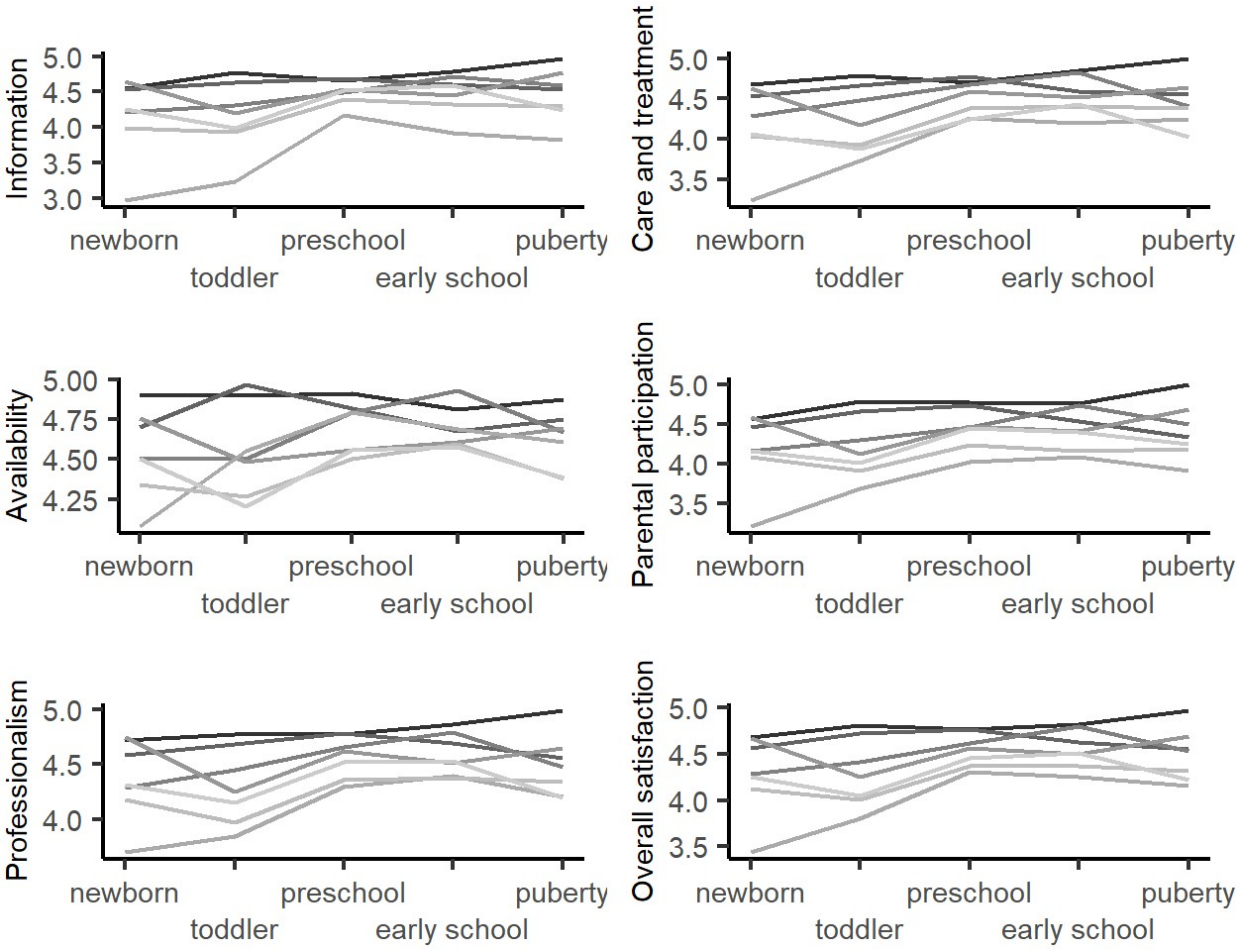
Trends of satisfaction in hospitals included in a study across child’s developmental stage. Notes: Separate lines represents seven different hospitals.

The children’s developmental stage significantly differentiated the overall parental satisfaction with care and satisfaction scores for the main domains: Information, Care and Treatment, Availability, Parental Participation and Professionalism. The parents of patients at the preschool, early school and puberty stage expressed the highest level of satisfaction with care. Trends of satisfaction in all domains across child’s developmental stage and by hospitals are depicted in Fig 3.

There were statistically significant differences between the reason of child’s hospitalisation and overall satisfaction level, as well as satisfaction in major criteria: Information, Parental Participation and Professionalism, and the lowest satisfaction scores were expressed by the parents of children admitted to hospital due to sudden illness.

Statistically significant differences were observed for both the overall parental satisfaction and satisfaction scores for all main domains (Information, Care and Treatment, Availability, Parental Participation and Professionalism) in terms of the children’s hospital stay length. The parents of children hospitalised for less than 7 and longer than 28 days demonstrated higher scores for both the overall satisfaction with care and satisfaction regarding all main domains compared to the parents of children with other hospital stay duration.

The results of multivariable analysis based on mixed effect regression with hospitals included as a random effect is shown in Table 5. After adjustment to multiple factors evident determinant of all considered domains of satisfaction was age of child. Regardless whether age was incorporated to the model as categorical variable (preschool/early school/puberty vs. newborn/infant/toddler) or as continuous, in all domains of satisfaction greater child’s age was associated with higher satisfaction of parents. However, in criterion Availability continuous analysis lost significance in model adjusted to all variables. The results indicates that achieving preschool age was very strong factor which increased assessment of satisfaction about 0.20–0.30 point on 1–5 point scale on average in most domain of satisfaction [β (95% CI): 0.23 (0.16; 0.31), R^2^ = 19% for overall satisfaction in Model 2]. Having more than 2 child significantly increased only Availability domain [β (95% CI): 0.15 (0.03; 0.27), R^2^ = 16% for overall satisfaction in Model 2]; see Table 5 and S1 and S2 Tables (supplementary tables present detailed results for all variables included); parents of children admitted to the hospital for diagnostic assessment or other reasons, estimated both higher level of overall satisfaction and satisfaction in major criteria such as Availability, Parental Participation and Professionalism. However in full adjusted models the result lost significance. We did not get a robust result for length of stay—the results were unstable depending on which covariates were included in the models.

**Table 5:** The association between selected demographic and hospital characteristic and parental satisfaction in each domain and in total—Multivariable regression.

Variable	Category	Information	Care and Treatment	Availability	Parental Participation	Professionalism	Overall satisfaction
**Child’s age**	**at least preschool/per year**						
	Model 1^&^	0.26 (0.16; 0.36)***	0.26 (0.16; 0.35)***	0.12 (0.03; 0.21)*	0.20 (0.10; 0.30)***	0.19 (0.11; 0.26)***	0.21 (0.12; 0.29)***
	Model 2^&^	0.29 (0.20; 0.39)***	0.28 (0.19; 0.37)***	0.15 (0.06; 0.23)**	0.21 (0.11; 0.30)***	0.20 (0.13; 0.28)***	0.23 (0.15; 0.30)***
	Model 3^#^	0.02 (0.01; 0.04)***	0.02 (0.01; 0.03)***	0.00 (-0.01; 0.02)	0.01 (0.00; 0.03)*	0.01 (0.00; 0.02)*	0.01 (0.01; 0.02)**
	Model 4^#^	0.03 (0.02; 0.04)***	0.02 (0.01; 0.03)***	0.01 (0.00; 0.02)*	0.02 (0.01; 0.03)**	0.01 (0.01; 0.02)***	0.02 (0.01; 0.03)***
**Children in family**	**2**						
	Model 1	-0.01 (-0.12; 0.09)	0.08 (-0.02; 0.18)	-0.01 (-0.11; 0.08)	-0.01 (-0.11; 0.1)	0.04 (-0.04; 0.13)	0.02 (-0.07; 0.10)
	Model 2	0.00 (-0.1; 0.10)	0.09 (-0.01; 0.19)	-0.01 (-0.10; 0.09)	-0.01 (-0.11; 0.1)	0.04 (-0.04; 0.12)	0.02 (-0.06; 0.11)
	Model 3	0.00 (-0.11; 0.10)	0.09 (-0.01; 0.19)	-0.02 (-0.11; 0.08)	-0.01 (-0.11; 0.1)	0.04 (-0.04; 0.13)	0.02 (-0.07; 0.11)
	Model 4	0.00 (-0.11; 0.10)	0.09 (-0.01; 0.19)	0.00 (-0.10; 0.09)	-0.01 (-0.11; 0.1)	0.04 (-0.04; 0.12)	0.02 (-0.06; 0.11)
	**>2**						
	Model 1	0.02 (-0.11; 0.15)	0.13 (0.00; 0.26)*	0.04 (-0.08; 0.16)	0.04 (-0.10; 0.17)	0.10 (0.00; 0.20)	0.06 (-0.04; 0.17)
	Model 2	0.03 (-0.09; 0.16)	0.15 (0.03; 0.27)*	0.04 (-0.07; 0.16)	0.05 (-0.08; 0.17)	0.09 (-0.01; 0.19)	0.07 (-0.03; 0.18)
	Model 3	0.04 (-0.10; 0.17)	0.14 (0.01; 0.27)*	0.02 (-0.10; 0.15)	0.03 (-0.10; 0.17)	0.10 (-0.01; 0.20)	0.06 (-0.05; 0.17)
	Model 4	0.03 (-0.10; 0.16)	0.15 (0.03; 0.28)*	0.05 (-0.07; 0.17)	0.05 (-0.08; 0.18)	0.10 (0.00; 0.20)	0.08 (-0.03; 0.18)
**Reason of admission**	**chronic disease exacerbation**						
	Model 1	0.01 (-0.09; 0.11)	0.00 (-0.09; 0.10)	0.04 (-0.05; 0.13)	0.01 (-0.09; 0.11)	0.04 (-0.03; 0.12)	0.02 (-0.06; 0.10)
	Model 2	0.07 (-0.07; 0.20)	0.01 (-0.11; 0.14)	-0.01 (-0.13; 0.11)	0.10 (-0.03; 0.23)	0.04 (-0.07; 0.14)	0.04 (-0.06; 0.15)
	Model 3	0.03 (-0.07; 0.14)	0.02 (-0.08; 0.12)	0.05 (-0.05; 0.14)	0.02 (-0.08; 0.12)	0.05 (-0.03; 0.13)	0.03 (-0.05; 0.12)
	Model 4	0.09 (-0.04; 0.22)	0.04 (-0.09; 0.16)	0.01 (-0.11; 0.13)	0.12 (-0.01; 0.25)	0.06 (-0.04; 0.16)	0.06 (-0.04; 0.17)
**Reason of admission**	**diagnostic or other**						
	Model 1	0.09 (-0.01; 0.20)	0.06 (-0.04; 0.16)	0.06 (-0.04; 0.15)	0.03 (-0.08; 0.13)	0.03 (-0.05; 0.12)	0.06 (-0.03; 0.14)
	Model 2	0.10 (-0.04; 0.25)	0.06 (-0.08; 0.19)	0.16 (0.03; 0.29)*	0.18 (0.04; 0.32)*	0.14 (0.03; 0.25)*	0.13 (0.01; 0.24)*
	Model 3	0.00 (-0.01; 0.01)	0.00 (0.00; 0.01)	0.01 (0.00; 0.02)*	0.00 (0.00; 0.01)	0.00 (0.00; 0.01)	0.00 (0.00; 0.01)
	Model 4	0.09 (-0.05; 0.24)	0.05 (-0.09; 0.18)	0.15 (0.02; 0.28)*	0.17 (0.03; 0.31)*	0.13 (0.02; 0.24)*	0.12 (0.00; 0.23)*
**Length of stay**	**8–28**						
	Model 1	0.23 (-1.02; 1.48)	0.11 (-0.89; 1.11)	0.13 (-0.3; 0.56)	0.22 (-0.91; 1.36)	0.20 (-0.62; 1.03)	0.18 (-0.75; 1.11)
	Model 2	-0.10 (-0.20; 0.01)	-0.08 (-0.18; 0.02)	-0.01 (-0.11; 0.09)	-0.03 (-0.14; 0.07)	-0.02 (-0.10; 0.06)	-0.05 (-0.13; 0.04)
	Model 3	0.25 (-0.97; 1.46)	0.13 (-0.85; 1.12)	0.15 (-0.27; 0.57)	0.24 (-0.87; 1.35)	0.22 (-0.58; 1.03)	0.2 (-0.71; 1.10)
	Model 4	-0.13 (-0.23; -0.02)*	-0.11 (-0.21; -0.01)*	-0.03 (-0.12; 0.07)	-0.06 (-0.16; 0.05)	-0.04 (-0.13; 0.04)	-0.07 (-0.16; 0.02)
	**>28**						
	Model 1	0.53 (-0.81; 1.87)	0.51 (-0.58; 1.61)	0.35 (-0.15; 0.84)	0.56 (-0.66; 1.79)	0.48 (-0.42; 1.37)	0.51 (-0.5; 1.51)
	Model 2	0.31 (0.02; 0.59)*	0.19 (-0.08; 0.46)	0.15 (-0.11; 0.41)	0.15 (-0.13; 0.43)	0.24 (0.02; 0.46)*	0.21 (-0.02; 0.44)
	Model 3	0.50 (-0.81; 1.80)	0.50 (-0.57; 1.58)	0.36 (-0.12; 0.85)	0.55 (-0.65; 1.75)	0.47 (-0.40; 1.35)	0.49 (-0.49; 1.47)
	Model 4	0.27 (-0.02; 0.55)	0.15 (-0.12; 0.42)	0.13 (-0.13; 0.39)	0.12 (-0.16; 0.40)	0.21 (-0.01; 0.43)	0.18 (-0.05; 0.41)
**R**^**2**^ **[range] [%]**		16.50–17.89	15.07–16.39	7.47–8.11	13.51–14.67	15.71–17.49	16.08–17.57

*p<0.05;

**p<0.001;

***p<0.0001;

^&^child’s age as categorical variable preschool/early school/puberty vs newborn/infant/toddler;

# child’s age as continuous variable result per year.

Notes:

**Model 1**: Child’s age [preschool/early school/puberty vs newborn/infant/toddler (ref^#^)], number of children in family [>2; 2 vs 1 (ref)], parent’s education [high vs other (ref)], parent’s age [more than 30 vs at most 30 (ref)], the level of health care coverage [Children’s hospital; 2nd level hospital; 3rd level hospital; Pulmonology/Oncological hospital vs Nationwide hospital (ref)], reason of admission [chronic disease exacerbation; diagnostic assessment and other vs sudden illness (ref)], length of stay [8–28 days; >28 vs ≤7 days (ref)];

**Model 2**: Child’s age [preschool/early school/puberty vs newborn/infant/toddler (ref)], number of children in family [>2; 2 vs 1 (ref)], reason of admission [chronic disease exacerbation; diagnostic assessment and other vs sudden illness (ref)], length of stay [8–28 days; >28 vs ≤7 days (ref)];

**Model 3**: Child’s age [continuous (per year)], number of children in family [>2; 2 vs 1 (ref)], parent’s education [high vs other (ref)], parent’s age [continuous (per year)], the level of health care coverage [Children’s hospital; 2nd level hospital; 3rd level hospital; Pulmonology/ Oncological hospital vs Nationwide hospital (ref)], reason of admission [chronic disease exacerbation; diagnostic assessment and other vs sudden illness], length of stay [8–28 days, >28, ≤7 days (ref)];

**Model 4**: Child’s age [continuous (per year)], number of children in family [>2; 2 vs 1 (ref)], reason of admission [chronic disease exacerbation; diagnostic assessment and other vs sudden illness (ref)], length of stay [8–28 days; >28 vs ≤7 days (ref)].

^#^ref—reference category

## Discussion

Assessment of patient satisfaction with services received is considered to be the backbone of high quality patient-centred care [21]. The essential factors in terms of providing patients with quality nursing care are identifying, understanding and meeting their expectations about care as well as considering the familial and social contexts in action planning [5, 22].

The results of studies conducted in various scientific centres using standardized research tools [2, 4, 5, 7, 10, 23–26] or original survey questionnaires [8, 27, 28] indicate the high level of parental satisfaction with nursing care or its specific aspects. Also in the current study most of parents were highly pleased with nursing care taking into account both overall satisfaction and all of major criteria.

As presented, more than 90% of respondents expressed high level of satisfaction with nurses’ Availability. A slightly lower, but still high level of satisfaction with nursing care was indicated in the criteria Parental Participation and Information, similarly to the findings of Smoleń and Ksykiewicz-Dorota [4] and Mol et al. [23]. According to scientific reports [24, 29, 30], the level of satisfaction with transfer of information is related to the parental satisfaction with participation in care and limited the parents’ claims in this issue [24]. Many authors point to the need for optimisation nursing services in both of these domains of care [7, 24, 25, 31–33]. It was proved that parents with a stronger involvement in the care about a child demonstrated a higher level of satisfaction of the overall stay in hospital, better understanding of information and confidence in the medical team, as well as lower level of anxiety [25, 31, 34]. Thus, improvement of care regarding information and participation of parents in the therapeutic process may lead to a higher level of satisfaction with the care about a child, shaping the skills and attitudes of parents necessary for better post-discharge care of their children and achieving better long-term treatment outcomes.

The results proved that the parents of children admitted to hospital on the elective basis demonstrated higher scores regarding satisfaction with care in terms of Information and Parental Participation compared to the parents of children with emergency admissions. These findings are partly consistent with the preliminary report of Kruszecka-Krówka et al. [2] where parents of elective patients expressed statistically significant higher satisfaction within nearly all care areas except Professional Attitude. However, according to the report provided by Smoleń and Ksykiewicz-Dorota [4], parents of children admitted to hospital in the emergency mode higher assessed the possibility of participating and decision-making regarding care than the parents of children with elective mode of admission.

A disease and disease-related hospital stay are difficult for a child, although the way of going through this time and reactions to hospitalisation vary, depending on the developmental stage, patients’ maturity level and individual factors [28]. The study revealed that the parents of patients at the preschool, early school and puberty stages expressed the highest overall satisfaction with care. The lowest scores regarding satisfaction with care were observed among toddlers’ parents. The child’s achievement of preschool age was the strongest indicator that increased the satisfaction rating in most domains. Other authors’ research also suggested a higher level of parental satisfaction with care among the parents of older children [2, 25, 26]. Specificity of early developmental stages determines the character of disease signs and symptoms, the scope of parental participation in care activities, the process of adaptation to hospitalisation and enhanced separation anxiety in children. The above factors may generate greater needs about nursing care among parents of younger children that affect the satisfaction level [2]. On the other hand, Willebrand et al. [24] and Sam et al. [35] observed different findings. However, it should be noted that the authors [24, 35] used another research instrument, the research was conducted in a very small group of parents whose children required specific surgical treatment and the parents did not accompany their children during the hospital stay [24].

The current study showed that the parents of children hospitalised for sudden illness expressed lower overall satisfaction level with care as well as lower satisfaction in major criteria: Information, Parental Participation and Professionalism compared to the parents of children admitted to hospital for other reasons. However, other authors’ findings did not reveal this relationship [4] or proved, that the subjective assessment of the child’s health by the parents and the level of the parents’ anxiety severity determined their satisfaction with nursing care, regardless of the reason for hospitalization [7, 24]. This would be a valuable aspect to be included into future research on satisfaction with care.

An important determinant of satisfaction with care was the length of children’s hospital stay, similarly to the research conducted by Smoleń & Ksykiewicz-Dorota [4, 10] and Divecha et al. [14]. The highest satisfaction with care or its selected areas was demonstrated by the parents of children hospitalised for less than 7 and longer than 28 days. It should be noted that a patient with increasingly longer hospitalisation time becomes better adapted to its environment. Parents also show gradual adaptation to the situation of their children’s disease and the therapeutic process. It can be assumed that the longest hospital stay, over 28 days, resulted markedly from a serious health condition of a child. Thus, a successful therapy could lead to a higher level of parental satisfaction, which was also observed in other study [7]. Hence, the determinants of respondents’ higher satisfaction could be satisfactory treatment outcomes and a positive degree of adaptation to the disease and the hospitalisation setting.

Scientific reports [7, 35, 36] suggest a limited influence of the socio-demographic parents’ variables on their satisfaction with the services provided throughout the hospital stay. Yet, the results of current study demonstrated that the sex, age, education and the number of children were important predictors of parental satisfaction with nursing care or its selected areas.

Different priorities and expectations about the child hospital care presented by men and women may determine differences in perception of the services received, which was confirmed by the study findings. The mothers showed a statistically significant higher satisfaction with nursing availability compared to the fathers. However, research findings in this field vary from those consistent with our outcomes [10], through absence of differences regarding the sex-related satisfaction levels [24, 26, 35], up to different findings observed [4, 7].

The parents aged less than 30 years expressed poorer satisfaction with the services provided compared to the parents in the other age groups. Less extensive life and parental experience may determine wide range of nursing care needs. This can be also explained by the fact, that having more than 2 child significantly increased parental satisfaction in Availability domain. Considering the fact that scientific findings in this field do not provide unequivocal opinions [4, 24, 25], it can be concluded that parental satisfaction, in addition to selected research instruments and the study group size, could be affected by the presence of other variables, not included in our own study as well.

Moreover, the lowest satisfaction level was seen among the parents with higher education, which partly complied with other authors findings [2, 4, 15, 25]. Yet, a group of researchers of the analysed scientific literature in the field of parental satisfaction with care do not prove the relationship between parents’ education levels and satisfaction with care [26, 35, 36] or suggest that parents with higher education placed more confidence in the competence of the medical team as well as expressed higher satisfaction with the support and information received [14].

To sum up, the analysis of scientific reports and the results of current study indicate that parents of hospitalised children were highly satisfied with nursing care. However, there are some points to improve and optimise, which are crucial both for the overall assessment of parental satisfaction with care and for achieving better long-term health outcomes in children. Differences between our study findings and those obtained by other authors may not only result from the selection of research instruments and the study group size, but also from parents’ expectations to be determined by the overall level of medical services in specific countries, financial aspects of the health care system, cultural factors as well as organisation and conditions of the nurse staff work, including their professional qualifications [37, 38]. Multifactorial conditions of parental satisfaction with nursing care inspire further studies in this field.

## Conclusions

Parents express a high level of general satisfaction with nursing services, and lower satisfaction of respondents concern especially their participation in care. There is a need for optimising nursing care especially in the area of parental participation. The institution’s level of health care coverage, the hospital admittance procedure, the child’s developmental stage, the cause and length of hospital stay as well as the age, education level of parents and the number of their children are important predictors of parental satisfaction with nursing care. The child’s achievement of preschool age is the strongest indicator that increase the satisfaction rating in most domains. The nursing care’ quality improvement plan in paediatric departments should focus particularly on early childhood patients and their parents who are the most critical in satisfaction’ assessment.

### Limitations and implications of research

The findings of this study, conducted in a representative group of parents, are the basis for development and implementation of the training plan for nursing teams in paediatric hospitals with the aim of optimisation of child nursing services regarding children and their parents/families.

Better quality services and higher parental satisfaction with care, particularly in the areas presented, may be the factors to reduce social costs of hospital treatment. The use of a standardised instrument to assess parental satisfaction and its implementation in the nursing practice may contribute to objectivization of other research findings in this field and unification of nursing service standards without affecting the personalised approach to patients.

Among the study limitations, too few participants in the study group consisting of parents of children hospitalised in surgical wards and lack of assessment at hospital admission of: parents’ expectations about nursing care, a child’s clinical status and parents’ perception of the patient’s health, should be mentioned. In addition, the study did not include the assessment of the respondents’ emotional states that could determine their needs regarding care and satisfaction with the services provided. The above limitations suggest a multidimensional aspect of the concept of satisfaction with nursing care and determine several directions of future research in this field.

## Supporting information

S1 Fig A graphical assessment of the goodness-of-fit of random-effects linear models—QQ-plots of the random effects. (TIF)

S2 Fig A graphical assessment of the goodness-of-fit of random-effects linear models—QQ-plots of the residuals. (TIF)

S1 Table The association between demographic and hospital characteristic and parental satisfaction in each domain and in total—Multivariable regression, the coefficients for all explanatory variables included in Model 1. p<0.05; **p<0.001; ***p<0.0001.
(DOC)

S2 Table The association between demographic and hospital characteristic and parental satisfaction in each domain and in total—Multivariable regression, the coefficients for all explanatory variables included in Model 3. p<0.05; **p<0.001; ***p<0.0001.
(DOC)

S1 File (DOCX)
