# Supplementary figures and images for: Selected predictors of parental satisfaction with child nursing care in paediatric wards in Poland—Cross-sectional study
Source: PLoS One. 2021 Nov 19;16(11):e0260504. doi: 10.1371/journal.pone.0260504 (PMC8604320; doi:10.1371/journal.pone.0260504)

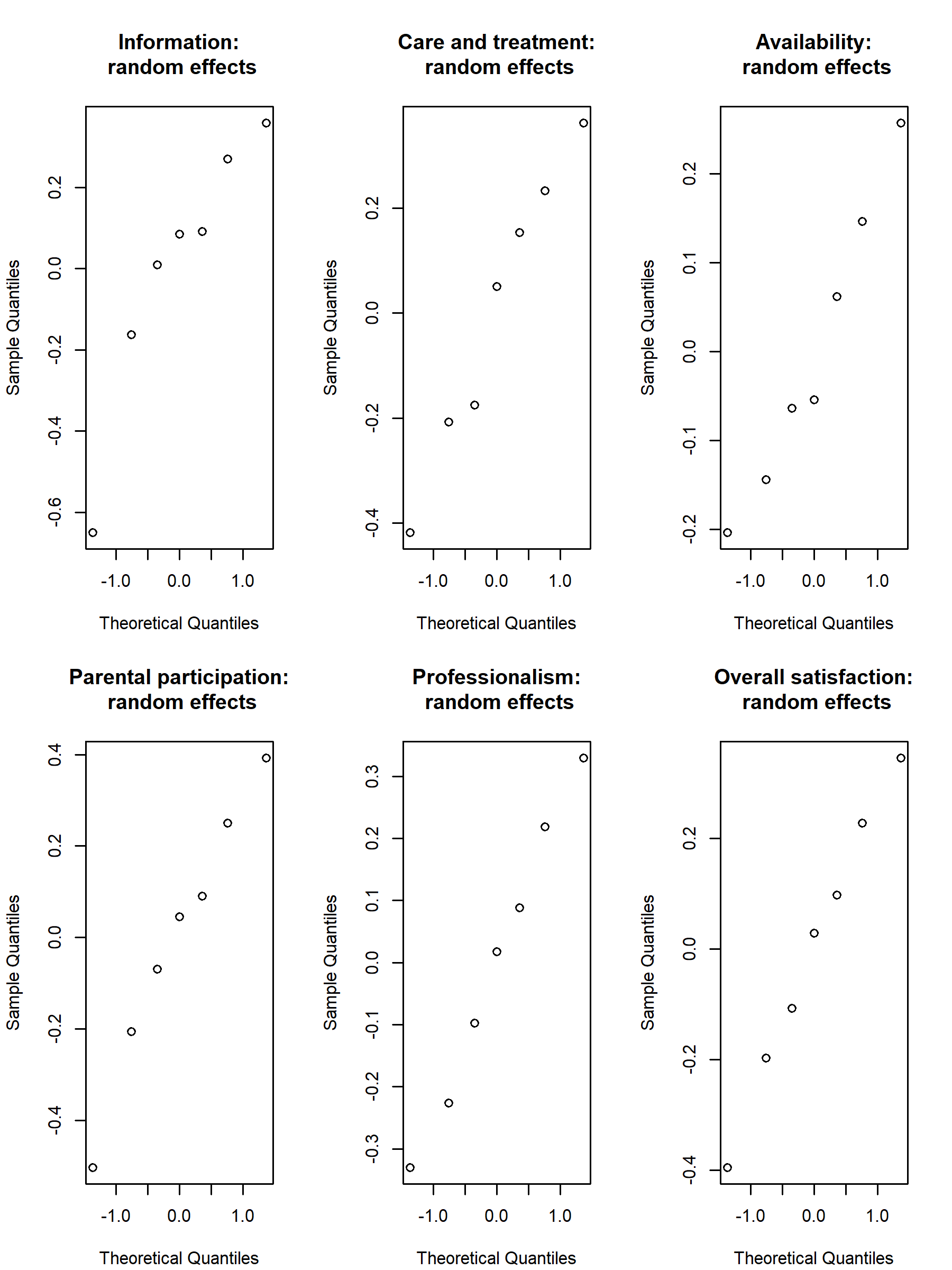

Supplement: S1 Fig — (TIF) [file pone.0260504.s001.tif]

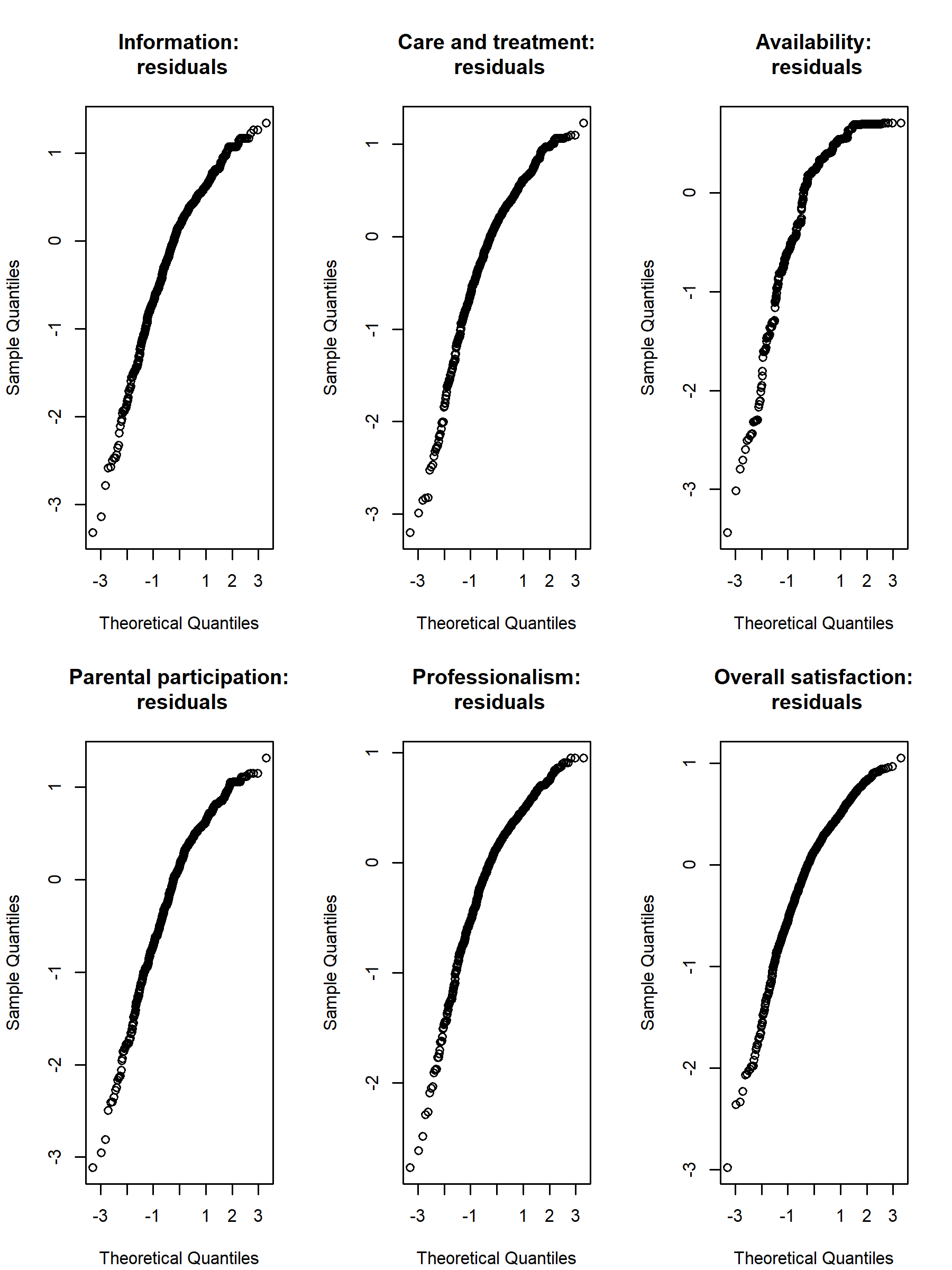

Supplement: S2 Fig — (TIF) [file pone.0260504.s002.tif]
