# Supplementary material for: Selected predictors of parental satisfaction with child nursing care in paediatric wards in Poland—Cross-sectional study
Source: PLoS One. 2021 Nov 19;16(11):e0260504. doi: 10.1371/journal.pone.0260504 (PMC8604320; doi:10.1371/journal.pone.0260504)
Supplement: S1 Table — p<0.05; **p<0.001; ***p<0.0001. (DOC) [file pone.0260504.s003.doc]

**S1 Table. The association between demographic and hospital characteristic and parental satisfaction in each domain and in total - multivariable regression, the coefficients for all explanatory variables included in Model 1.**

| **Variable** | **Category** | **Information** | **Care and Treatment** | **Availability** | **Parental**  **Participation** | **Professionalism** | **Overall satisfaction** |
| --- | --- | --- | --- | --- | --- | --- | --- |
| **Sex** | male | -0.05 (-0.19; 0.09) | -0.04 (-0.17; 0.09) | -0.02 (-0.15; 0.10) | -0.05 (-0.19; 0.09) | -0.01 (-0.11; 0.10) | -0.03 (-0.15; 0.08) |
| **Child's age** | at least preschool | 0.26 (0.16; 0.36)*** | 0.26 (0.16; 0.35)*** | 0.12 (0.03; 0.21)* | 0.20 (0.10; 0.30)*** | 0.19 (0.11; 0.26)*** | 0.21 (0.12; 0.29)*** |
| **Children in family** | 2 | -0.01 (-0.12; 0.09) | 0.08 (-0.02; 0.18) | -0.01 (-0.11; 0.08) | -0.01 (-0.11; 0.10) | 0.04 (-0.04; 0.13) | 0.02 (-0.07; 0.10) |
|  | >2 | 0.02 (-0.11; 0.15) | 0.13 (0.00; 0.26)* | 0.04 (-0.08; 0.16) | 0.04 (-0.10; 0.17) | 0.10 (0.00; 0.20) | 0.06 (-0.04; 0.17) |
| **Education** | high | 0.01 (-0.09; 0.11) | 0.00 (-0.09; 0.10) | 0.04 (-0.05; 0.13) | 0.01 (-0.09; 0.11) | 0.04 (-0.03; 0.12) | 0.02 (-0.06; 0.10) |
| **Parent's age** | >30 | 0.09 (-0.01; 0.20) | 0.06 (-0.04; 0.16) | 0.06 (-0.04; 0.15) | 0.03 (-0.08; 0.13) | 0.03 (-0.05; 0.12) | 0.06 (-0.03; 0.14) |
| **Hospital level** | Children’s hospital | 0.23 (-1.02; 1.48) | 0.11 (-0.89; 1.11) | 0.13 (-0.30; 0.56) | 0.22 (-0.91; 1.36) | 0.20 (-0.62; 1.03) | 0.18 (-0.75; 1.11) |
|  | 2nd level hospital | 0.53 (-0.81; 1.87) | 0.51 (-0.58; 1.61) | 0.35 (-0.15; 0.84) | 0.56 (-0.66; 1.79) | 0.48 (-0.42; 1.37) | 0.51 (-0.50; 1.51) |
|  | 3rd level hospital | -0.12 (-1.34; 1.10) | 0.08 (-0.91; 1.07) | 0.33 (-0.09; 0.76) | 0.04 (-1.07; 1.16) | 0.19 (-0.62; 1.00) | 0.09 (-0.82; 1.01) |
|  | Pulm./Oncol. hospital^&^ | 0.45 (-0.99; 1.90) | 0.44 (-0.73; 1.60) | 0.41 (-0.09; 0.91) | 0.49 (-0.83; 1.80) | 0.48 (-0.47; 1.44) | 0.46 (-0.62; 1.54) |
| **Reason of admission** | chronic disease exacerbation | 0.04 (-0.09; 0.18) | -0.01 (-0.14; 0.12) | -0.03 (-0.15; 0.10) | 0.07 (-0.06; 0.21) | 0.02 (-0.08; 0.13) | 0.02 (-0.09; 0.13) |
|  | diagnostic or other | 0.11 (-0.04; 0.25) | 0.06 (-0.08; 0.20) | 0.17 (0.04; 0.30)* | 0.18 (0.04; 0.32)* | 0.14 (0.03; 0.25)* | 0.13 (0.02; 0.25)* |
| **Length of stay** | 8-28 | -0.09 (-0.2.00; 0.02) | -0.07 (-0.18; 0.03) | 0.00 (-0.10; 0.10) | -0.03 (-0.14; 0.07) | -0.01 (-0.10; 0.07) | -0.04 (-0.13; 0.05) |
|  | >28 | 0.31 (0.02; 0.6.00)* | 0.2 (-0.08; 0.47) | 0.16 (-0.11; 0.42) | 0.18 (-0.10; 0.47) | 0.25 (0.03; 0.48)* | 0.22 (-0.01; 0.45) |
| **Variance** | hospital | 0.27 (33.95%) | 0.17 (26.44%) | 0.03 (6.23%) | 0.22 (30.10%) | 0.12 (27.02%) | 0.15 (30.40%) |
|  | residual | 0.52 (66.05%) | 0.48 (73.56%) | 0.44 (93.77%) | 0.51 (69.90%) | 0.31 (72.98%) | 0.34 (69.60%) |
|  | **R2 [%]** | 17.89 | 16.39 | 8.11 | 14.67 | 17.49 | 17.57 |

*p<0.05; **p<0.001; ***p<0.0001; ^&^Pulm/Onkol hospital - Pulmonology/Oncological hospital

Notes: child’s age [preschool/early school/puberty vs newborn/infant/toddler (ref^#^)], number of children in family [>2; 2 vs 1 (ref)], parent’s education [high vs other (ref)], parent’s age [more than 30 vs at most 30 (ref)], the level of health care coverage [Children’s hospital; 2nd level hospital; 3rd level hospital; Pulmonology/Oncological hospital vs Nationwide hospital (ref)], reason of admission [chronic disease exacerbation; diagnostic assessment and other vs sudden illness (ref)], length of stay [8-28 days; >28 vs ≤7 days (ref)];

^#^ref - reference category
