# Supplementary material for: Selected predictors of parental satisfaction with child nursing care in paediatric wards in Poland—Cross-sectional study
Source: PLoS One. 2021 Nov 19;16(11):e0260504. doi: 10.1371/journal.pone.0260504 (PMC8604320; doi:10.1371/journal.pone.0260504)
Supplement: S2 Table — p<0.05; **p<0.001; ***p<0.0001. (DOC) [file pone.0260504.s004.doc]

**S2 Table. The association between demographic and hospital characteristic and parental satisfaction in each domain and in total - multivariable regression, the coefficients for all explanatory variables included in Model 3.**

| **Variable** | **Category** | **Information** | **Care and Treatment** | **Availability** | **Parental Participation** | **Professionalism** | **Overall satisfaction** |
| --- | --- | --- | --- | --- | --- | --- | --- |
| **Sex** | male | -0.03 (-0.17; 0.11) | -0.02 (-0.16; 0.11) | -0.02 (-0.15; 0.10) | -0.04 (-0.18; 0.10) | 0.00 (-0.11; 0.11) | -0.02 (-0.14; 0.09) |
| **Child's age** | continuous | 0.02 (0.01; 0.04)*** | 0.02 (0.01; 0.03)*** | 0.00 (-0.01; 0.02) | 0.01 (0.00; 0.03)* | 0.01 (0.00; 0.02)* | 0.01 (0.01; 0.02)** |
| **Children in family** | 2 | 0.00 (-0.11; 0.10) | 0.09 (-0.01; 0.19) | -0.02 (-0.11; 0.08) | -0.01 (-0.11; 0.10) | 0.04 (-0.04; 0.13) | 0.02 (-0.07; 0.11) |
|  | >2 | 0.04 (-0.10; 0.17) | 0.14 (0.01; 0.27)* | 0.02 (-0.10; 0.15) | 0.03 (-0.10; 0.17) | 0.10 (-0.01; 0.20) | 0.06 (-0.05; 0.17) |
| **Education** | high | 0.03 (-0.07; 0.14) | 0.02 (-0.08; 0.12) | 0.05 (-0.05; 0.14) | 0.02 (-0.08; 0.12) | 0.05 (-0.03; 0.13) | 0.03 (-0.05; 0.12) |
| **Parent's age** | continuous | 0.00 (-0.01; 0.01) | 0.00 (0.00; 0.01) | 0.01 (0.00; 0.02)* | 0.00 (0.00; 0.01) | 0.00 (0.00; 0.01) | 0.00 (0.00; 0.01) |
| **Hospital level** | Children’s hospital | 0.25 (-0.97; 1.46) | 0.13 (-0.85; 1.12) | 0.15 (-0.27; 0.57) | 0.24 (-0.87; 1.35) | 0.22 (-0.58; 1.03) | 0.20 (-0.71; 1.10) |
|  | 2nd level hospital | 0.50 (-0.81; 1.80) | 0.50 (-0.57; 1.58) | 0.36 (-0.12; 0.85) | 0.55 (-0.65; 1.75) | 0.47 (-0.40; 1.35) | 0.49 (-0.49; 1.47) |
|  | 3rd level hospital | -0.07 (-1.26; 1.12) | 0.12 (-0.85; 1.09) | 0.35 (-0.07; 0.77) | 0.08 (-1.01; 1.17) | 0.22 (-0.57; 1.01) | 0.13 (-0.76; 1.02) |
|  | Pulm./Oncol. hospital^&^ | 0.48 (-0.93; 1.88) | 0.46 (-0.68; 1.60) | 0.42 (-0.07; 0.91) | 0.51 (-0.78; 1.79) | 0.50 (-0.43; 1.43) | 0.48 (-0.57; 1.52) |
| **Reason of admission** | chronic disease exacerbation | 0.07 (-0.07; 0.21) | 0.01 (-0.11; 0.14) | -0.02 (-0.15; 0.10) | 0.09 (-0.05; 0.22) | 0.04 (-0.07; 0.14) | 0.04 (-0.07; 0.15) |
|  | diagnostic or other | 0.09 (-0.05; 0.24) | 0.05 (-0.09; 0.19) | 0.16 (0.03; 0.29)* | 0.17 (0.03; 0.32)* | 0.14 (0.03; 0.25)* | 0.12 (0.01; 0.24)* |
| **Length of stay** | 8-28 | -0.12 (-0.23; -0.01)* | -0.10 (-0.21; 0.00) | -0.02 (-0.12; 0.08) | -0.06 (-0.16; 0.05) | -0.04 (-0.12; 0.05) | -0.07 (-0.15; 0.02) |
|  | >28 | 0.27 (-0.02; 0.56) | 0.16 (-0.12; 0.44) | 0.14 (-0.13; 0.40) | 0.15 (-0.13; 0.44) | 0.23 (0.00; 0.45)* | 0.19 (-0.04; 0.42) |
| **Variance** | hospital | 0.25 (32.34%) | 0.17 (25.42%) | 0.03 (5.95%) | 0.21 (28.90%) | 0.11 (25.76%) | 0.14 (28.92%) |
|  | residual | 0.53 (67.66%) | 0.49 (74.58%) | 0.44 (94.05%) | 0.52 (71.10%) | 0.32 (74.24%) | 0.35 (71.08%) |
|  | **R2 [%]** | 16.69 | 15.14 | 7.98 | 13.96 | 16.42 | 16.43 |

*p<0.05; **p<0.001; ***p<0.0001; ^&^Pulm/Onkol hospital - Pulmonology/Oncological hospital.

Notes: child’s age [continuous (per year)], number of children in family [>2; 2 vs 1 (ref^#^)], parent’s education [high vs other (ref)], parent’s age [continuous (per year)], the level of health care coverage [Children’s hospital; 2nd level hospital; 3rd level hospital; Pulmonology/ Oncological hospital vs Nationwide hospital (ref)], reason of admission [chronic disease exacerbation; diagnostic assessment and other vs sudden illness], length of stay [8-28 days, >28, ≤7 days (ref)];

^#^ref - reference category
